# Huge racemose hemangioma of the bronchial artery with arterial supply via the right coronary artery and left internal thoracic artery

**DOI:** 10.1002/rcr2.1048

**Published:** 2022-09-29

**Authors:** Rikako Horie, Kensuke Sekiya, Junichi Funada

**Affiliations:** ^1^ Cardiology Ehime Medical Center Toon Japan

**Keywords:** angiography, bronchial artery, bronchial Dieulafoy's disease, coronary steal phenomenon, racemose hemangioma

## Abstract

A 79‐year‐old male with bronchiectasis was referred to our clinic because of mild chest tightness on exertion. He had no history of hemoptysis. An electrocardiogram showed ST segment depression in leads V5‐6. Multi‐detector contrast‐enhanced computed tomography revealed no significant stenosis in either coronary artery; however, a huge racemose hemangioma of the bronchial artery (RHBA) was detected. In addition, arterial supply to the RHBA via the right coronary artery (RCA) and the left internal thoracic artery (LITA) was suspected. Adenosine‐loading myocardial scintigraphy images revealed segmental hypo‐perfusion in the left ventricular inferior wall. Selective bronchial artery angiography revealed the huge RHBA. In addition, both the RCA and LITA provided arterial supply to the RHBA. To the best of our knowledge, this case is the first to show multiple arterial supply resulting in a huge RHBA.

## Graphical abstract

We present the first case of a huge racemose hemangioma of the bronchial artery (RHBA) with multiple arterial supply, and the clinical findings indicated the possibility of coronary steal phenomenon due to the arterial supply from the right coronary artery (RCA) to the hemangioma.

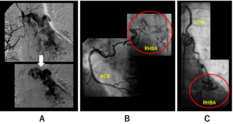

## INTRODUCTION

A racemose hemangioma of the bronchial artery (RHBA), also described as bronchial Dieulafoy's disease (BDD), is a rare abnormality showing dilated, tortuous bronchial arteries, with patients also showing respiratory‐related symptoms in most cases.^1^, ^2^ The aetiology and pathogenesis of RHBA are uncertain but include congenital vascular malformations or chronic bronchial injury secondary to previous pulmonary infections and inflammation. Therefore, respiratory‐related symptoms such as bloody sputum, hemoptysis, or cough are the most common symptoms of RHBA patients. In terms of the arterial source of RHBA, a previous report presented a dilated bronchial artery in 48 cases and a tortuous dilated pulmonary artery in 4 cases out of 52 RHBA patients.^1^ This case demonstrates a huge RHBA with multiple arterial supply including the right coronary artery (RCA), with the patient showing mild chest oppression on effort.

## CASE REPORT

**FIGURE 1:**
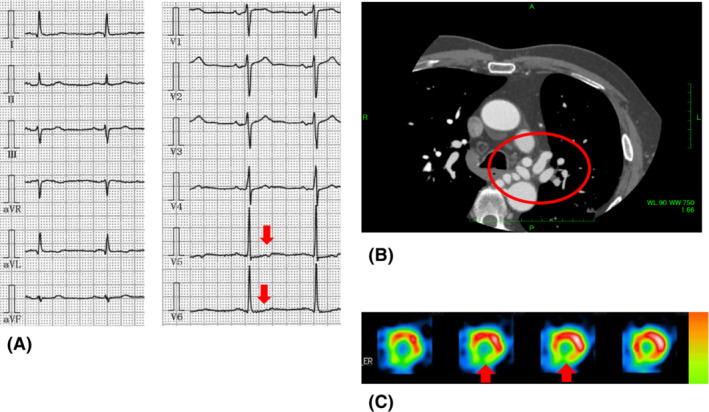
(A) An electrocardiogram showing ST depression in leads V5, 6 (red arrows). (B) Multi‐detector contrast‐enhanced computed tomography (CCT) showing accumulation of enhanced vasculatures (red circle), indicating a racemose hemangioma of the bronchial artery. (C) Adenosine‐loading myocardial scintigraphy showing hypo‐perfusion in the inferior wall of the left ventricle (red arrows)

**FIGURE 2:**
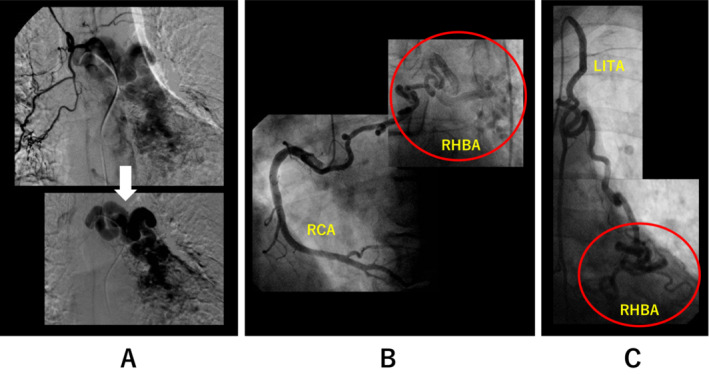
Selective bronchial artery angiography (A, digital subtraction image) showing the main body of the huge racemose hemangioma of the bronchial artery (RHBA). Right coronary artery (RCA) and left internal thoracic artery (LITA) angiography images showing the arterial supply to the RHBA (B, C)

A 79‐year‐old male was referred to the department of cardiology to investigate the cause of mild chest discomfort on effort. He had undergone regular medical check‐ups for bronchiectasis at the department of respiratory medicine. An electrocardiogram revealed ST depression in leads V5‐6 (Figure 1A). Multi‐detector contrast‐enhanced computed tomography (CCT) revealed no significant stenosis in either coronary artery; however, a huge RHBA was detected (Figure 1B). In addition, arterial supply to the RHBA via the RCA and the left internal thoracic artery (LITA) was suspected. Adenosine‐loading myocardial scintigraphy revealed segmental hypo‐perfusion in the inferior wall of the left ventricle (Figure 1C). Selective bronchial artery angiography revealed the huge RHBA (Figure 2A). Coronary angiography revealed no significant stenosis in both coronary arteries; however, a well‐developed conus branch of RCA provided arterial supply to the RHBA (Figure 2B). Furthermore, selective LITA angiography revealed an additional arterial supply to the RHBA (Figure 2C) Because his chest symptom was spontaneously relieved and he had no history of clinically relevant bronchial bleeding, we did not perform a surgical intervention or arterial embolization but chose regular medical check‐ups instead. More than 6 years have passed without any clinical events related to RHBA.

## DISCUSSION

RHBA, also described as BDD, is a rare abnormality showing dilated, tortuous bronchial arteries, with patients also showing respiratory‐related symptoms in most cases.^1^, ^2^ The aetiology and pathogenesis of RHBA are uncertain but the disease has been classified into two types, congenital vascular malformations as a primary form and chronic bronchial injury related to previous pulmonary infections and inflammation as a secondary form.^1^, ^2^, ^3^ Our case could be regarded as a secondary form because the patient has undergone regular medical check‐ups for bronchiectasis. On the other hand, arterial supply from the RCA and LITA to a RHBA is a possible background of congenital vascular malformation. The combination of both primary and secondary forms resulted in a huge RHBA in this case. Qian et al. reviewed 73 cases of RHBA reported from 1995 to 2019.^1^ The arterial supply was mainly provided by bronchial arteries and only four cases presented the arterial source of RHBA via the pulmonary artery. Neither coronary arteries nor LITA was shown to be the source of RHBA in previous reviews.^1^, ^2^

The clinical symptoms of RHBA are non‐specific, but most patients show symptoms related to respiratory diseases such as bloody sputum, hemoptysis, cough, infection or respiratory failure.^1^, ^2^ In addition, recurrent hemoptysis is a common and critical symptom of RHBA. On the other hand, only one patient was reported to have chest pain as a clinical manifestation.^2^ Our patient complained of atypical chest oppression without clinically relevant respiratory‐related symptoms and hemoptysis. The cause of chest oppression in this case is uncertain; however, Coronary steal phenomenon of the RCA due to a feeding artery of RHBA derived from a well‐developed conus branch of the RCA might be an explanation. Myocardial ischemia in the left ventricular inferior wall revealed by adenosine‐loading myocardial scintigraphy supports our hypothesis. A previous report described the usefulness of the fractional flow reserve by a sensor wire to evaluate the coronary steal phenomenon in a patient with coronary‐pulmonary fistulas.^4^ A trans‐catheter physiological assessment of the RCA using a sensor wire should be considered if chest oppression recurs in this case.

Existing methods of treating RHBA include conservative internal medicine, bronchial arterial ligation, surgical lobectomy and bronchial artery embolization (BAE), although a typical treatment has not yet been established.^1^, ^2^ Historically, RHBA, especially in symptomatic patients, was treated surgically by lobectomy and ligation of the bronchial artery. A recent review demonstrated the advantages of BAE from the view point of minimal invasiveness, quickness and preservation of lung function.^2^ However, Qian et al. demonstrated that 52.6% of patients who underwent BAE as an initial management required surgical lobectomy due to a technical failure of BAE or a subsequent recurrence.^1^ There are no definite prognostic guidelines for asymptomatic RHBA. We chose regular medical check‐ups and subsequently, more than 6 years have passed without any clinical events related to RHBA. A recent review and a clinical case report also presented a relatively good outcome with conservative internal medicine.^2^, ^5^ Meticulous attention in this case should ensure that any clinical symptoms or the opportunity for therapeutic intervention will not be missed.

We discovered a huge asymptomatic RHBA in a patient with bronchiectasis. Both the RCA and LITA provided arterial supply to the RHBA. To the best of our knowledge, this case is the first to show multiple arterial supply including via the coronary artery to an RHBA.

## AUTHOR CONTRIBUTIONS

Rikako Horie and Junichi Funada made contributions to the conception and design of the work. Kensuke Sekiya contributed to the revision of the manuscript drafts. All authors have approved the submitted version of the manuscript and agreed to be accountable for any part of the work.

## CONFLICT OF INTEREST

None declared.

## ETHICS STATEMENT

The authors declare that appropriate written informed consent was obtained for the publication of this manuscript and accompanying images.

## Data Availability

The data that support the findings of this study are available from the corresponding author upon reasonable request.
